# Risk factors for coexisting deep endometriosis for patients with recurrent ovarian endometrioma

**DOI:** 10.3389/fsurg.2022.963686

**Published:** 2022-11-02

**Authors:** Yongjiang Du, Changchang Hu, Chaoshuang Ye, Ruijin Wu

**Affiliations:** Department of Obstetrics & Gynecology, Women’s Hospital, School of Medicine, Zhejiang University, Hangzhou, China

**Keywords:** recurrent ovarian endometrioma, deep endometriosis, risk factor, uterine retroversion, follow-up time

## Abstract

**Aim:**

The aim of this study was to assess the risk factors for coexisting deep endometriosis (DE) in patients with recurrent ovarian endometrioma (OE).

**Methods:**

We retrospectively reviewed 151 recurrent OE patients who had been diagnosed of OE but not DE at the time of their first surgery and then received a second surgery for recurrent endometriosis with or without DE. Their clinical characteristics at the time of the first and second surgeries were collected. Univariate and multivariate logistic regression analyses were conducted to identify potential risk factors for coexisting DE in patients with recurrent OE.

**Results:**

Among the 151 recurrent OE patients, 46 were diagnosed of DE during the recurrent surgery and included in the DE group, while the remaining 105 patients were included in the non-DE group. In univariate analysis, there were significant differences in terms of uterine retroversion during the primary surgery and the follow-up time after the primary surgery between the DE and non-DE groups. The multivariate analysis also showed that both uterine retroversion and the follow-up time (≥5 years) were associated with the coexistence of DE during the recurrent surgery. The odds ratio (OR) for uterine retroversion was 3.72 [95% confidence interval (CI) 1.62–8.53], and the OR for follow-up time (≥5 years) was 5.03 (95% CI 2.29–11.02).

**Conclusions:**

Our study suggested that for recurrent OE patients, uterine retroversion during the first surgery and a follow-up time of at least 5 years are risk factors for the coexistence of DE in recurrent surgery, early prevention and full preparation before the recurrent surgery should be emphasized in these conditions.

## Introduction

Endometriosis is a chronic condition that affects as many as 10% of women of reproductive age (1). Retrograde menstruation, coelomic metaplasia, and lymphatic and vascular metastasis are the most common theoretical explanations for the origin of extra-uterine endometriotic tissue, and the development of endometriosis may involve interacting endocrine, immunological, proinflammatory, and proangiogenic processes (2). There are three types of endometriotic lesions: peritoneal endometriosis (PE), ovarian endometrioma (OE), and deep endometriosis (DE). It was suggested long ago that they may represent three clinically separate disease entities with different pathogenesis (3). According to the genetic/epigenetic theory, the development and maturation of lesions into PE, OE, or DE was postulated to be a consequence of the genetic background as well as the local environmental disturbances (4), and previous studies have already shown that several of the DE pathogenetic features are specific in comparison to other endometriosis phenotypes (5–7).

Unlike OE, which can be diagnosed by transvaginal ultrasound alone with a sensitivity of 90% and a specificity of 97% (8), DE is difficult to diagnose, and there is often a considerable delay (9). The treatment of DE is also a thorny problem. Medical approaches may control but not eradicate DE, while surgical approaches can be risky, and treated at referral centers that have the knowledge and experience for DE is often recommended. Thus, knowing the risk factors for developing DE is of great importance.

Most DE lesions present with other forms of endometriosis, and about half DE lesions present with OE (10). It is estimated that DE can affect 20% of women with pelvic endometriosis (9). However, in recurrent endometriosis, the incidence of DE was reported to be even higher (11). Until now, few studies have reported the incidence and risk factors of coexisting DE in recurrent OE patients. Therefore, in the present study, we aimed to investigate the potential risk factors for the development of DE lesions among recurrent OE patients.

## Materials and methods

Data were obtained from patients who were diagnosed and treated for recurrent endometriosis between January 2008 and December 2019 at the Women's Hospital, School of Medicine, Zhejiang University. The study protocol was approved by the local Ethics Committee (no. 2019–328).

The inclusion criteria were as follows: (1) patients of reproductive age who had a primary and a second surgery for endometriosis with laparoscopy, both operated in our hospital; (2) patients who had been diagnosed of OE but not DE at the time of the first surgery; (3) patients who had been diagnosed of recurrent OE coexisted with or without DE and underwent a second surgery for recurrence; and (4) all diagnoses were confirmed by histopathological examination. The exclusion criteria were as follows: (1) patients who underwent semiradical procedures, such as hysterectomy or oophorectomy during the first surgery; (2) during the first surgery, patients who had the following conditions: deep dyspareunia, severe gastrointestinal symptoms, severe dysmenorrhea, infertility, cul-de-sac obliteration, or adenomyosis, which may indicate the coexistence of DE (11–13); and (3) patients who had other concomitant malignant diseases found during the first surgery. DE was defined as (14, 15): (1) the muscularis of the bladder, the intestine, or the intrinsic ureter was infiltrated by endometriotic tissue after radical surgery (e.g., bowel resection, partial cystectomy, and ureteral resection); and (2) endometriotic tissue infiltrated beneath the peritoneum surface deeper than 5 mm in other locations, such as the uterosacral ligament(s), the vagina, or the extrinsic ureter.

The initial surgeries for OE were performed with laparoscopy by experienced gynecologists. After inspection of the pelvic and peritoneal organs, the disease staging was estimated. Adhesions were separated by blunt and sharp dissection. After the cyst content was aspirated, the cyst capsule was thoroughly stripped from the normal ovarian tissue. PE lesions were coagulated.

Patients were offered postoperative hormonal treatment to prevent recurrence based on availability and after fully informed consent was obtained. Postoperative hormonal treatments included gonadotropin-releasing hormone agonist (GnRH-a), progestin (P) or combined oral contraceptives (COC), or a levonorgestrel-releasing intrauterine system (LNG-IUS). GnRH-a was given periodically (leuprorelin or triptorelin acetate 3.75 mg subcutaneous injection every 4 weeks), or the LNG-IUS was inserted into the uterine cavity by the surgeon during the first surgery or after the patient finished GnRH-a treatment or other medical treatments.

Patients were followed up every 6–12 months. The indications for recurrent surgery were as follows (16–18): (1) medical treatment is ineffective for the associated pain; (2) patients have infertility; (3) the cyst is rapidly growing or is suspected to be malignant; (4) bowel, urinary obstruction or dysfunction; and (5) women who declined or had contraindications to the use of hormones. The follow-up time refers to the period from the first surgery to the second surgery. During the second surgery, DE was treated by shaving, disc excision, bowel resection or other kinds of techniques according to the specific location of the disease, and hysterectomy or oophorectomy was performed according to the severity of the disease. The second surgeries were performed by experienced and qualified gynecologists or surgeons.

Clinical data such as age, gravida, parity, dysmenorrhea, dyspareunia, infertility, largest diameter of ovarian endometrioma, uterine retroversion during the first surgery, medical treatment duration after the first surgery, and the follow-up time were collected. Statistical analysis was performed using SPSS 20 software (SPSS Inc., Chicago, IL, USA), with statistical significance being accepted at *p* < 0.05. Mann–Whitney U tests or Pearson's chi-square tests were performed for quantitative or qualitative variables, as appropriate, in the univariate analysis. The variables with *p* < 0.2 in the univariate analysis were introduced into the multivariate analysis. Odds ratios (ORs) with 95% confidence intervals (CIs) were calculated using logistic regression analysis.

## Results

**Figure 1:**
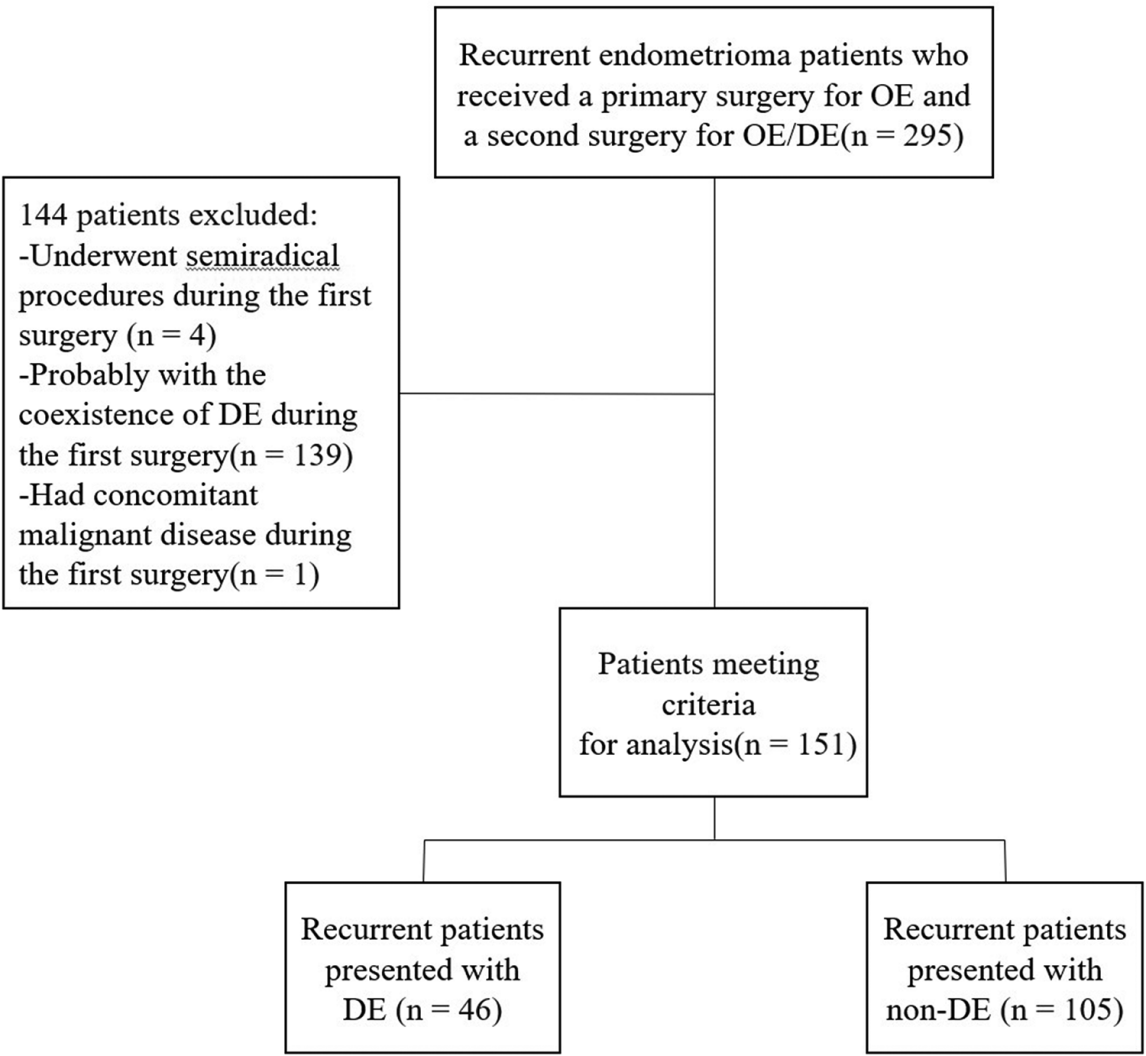
Flow diagram of patient selection process.

A total of 151 recurrent OE patients were included in this study. Among them, 46 were diagnosed of coexisting DE and thus classified as DE group, the remaining patients were classified as non-DE group. A flow chart of the current study is shown in Figure 1.

**Figure 2:**
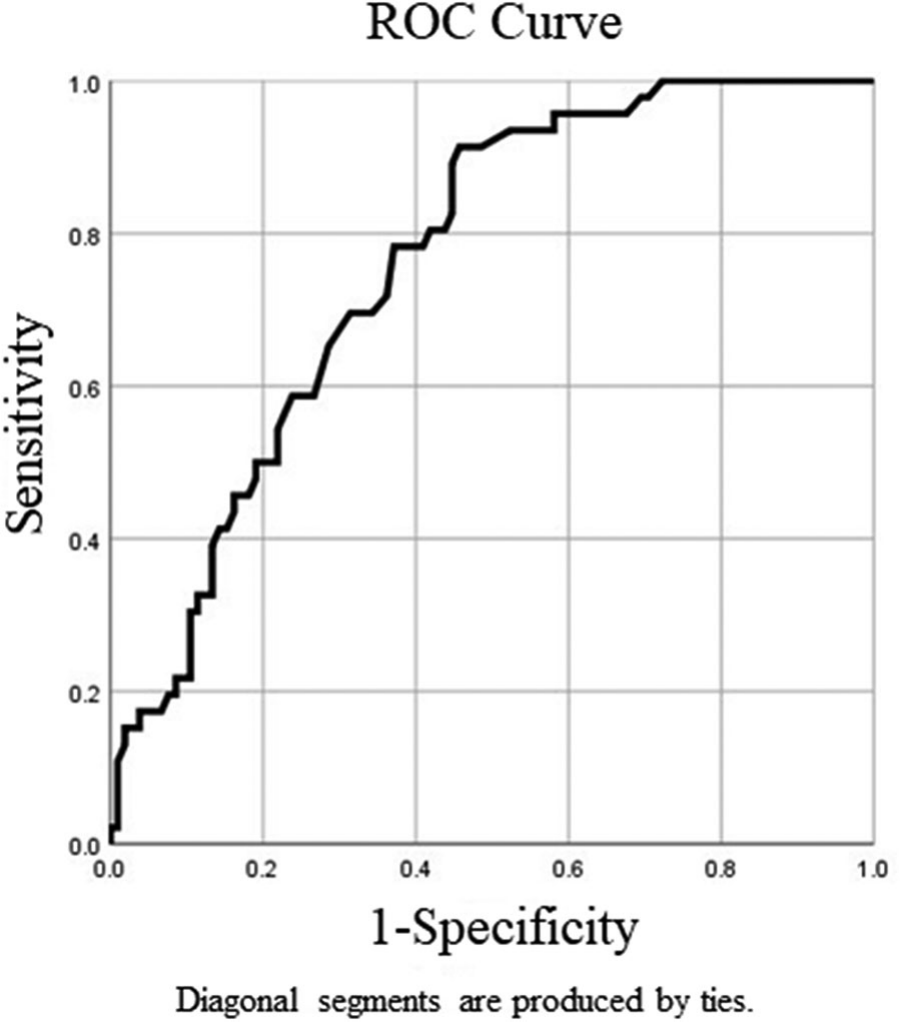
ROC curve analysis of follow-up time for deep endometriosis.

Clinical information at the time of the first surgery and during the follow-up periods as well as those factors that were considered as putative risk factors for coexisting DE for patients with recurrent OE is recorded in Table 1. During the first surgery, there was no significant difference in terms of age, body mass index (BMI), gravidity, parity, CA125 level, largest cyst diameter, or postoperative treatment duration in the univariate analysis between the DE group and the non-DE group. Twenty-one out of 46 patients (46%) had uterine retroversion in the DE group, while 21 out of 105 patients (20%) had uterine retroversion in the non-DE group, and there was a significant difference in the univariate analysis between the two groups (*p* < 0.01). The mean follow-up time was 82.7 months in the DE group and 51 months in the non-DE group, and there was also a significant difference between the two groups (*p* < 0.01) (Table 1). ROC curve analysis was performed to detect the optimum cutoff value of the follow-up time for the presence of DE (Figure 2). In the ROC curve analysis, the AUC (95% CI) value of the follow-up time was 0.762 (0.686–0.838). In addition, 63.5 months was found to be the optimum cutoff value according to the Youden index criteria. For the convenience of clinical applications, we set 5 years (60 months) as the cutoff value and defined a follow-up time of at least 5 years as a long follow-up time. During the first 5 years after the primary OE surgery, 16 out of 91 (18%) recurrent patients who did not have a long follow-up time developed DE. However, when starting 5 years after the primary surgery, 30 out of 60 (50%) recurrent patients who had a longer follow-up time finally developed DE, the probability of coexistence of DE in recurrent surgery had almost tripled. The multivariate analysis also showed a statistically significant high OR of uterine retroversion (3.72; 95% CI 1.62–8.53) and that of a long follow-up time (5.03; 95% CI 2.29–11.02) for the presence of DE (Table 2).

**Table 1:** Comparison of clinical characteristics during the first surgery and the follow-up periods .

Variable	DE group (*n* = 46)	Non-DE group (*n* = 105)	*p* value
Age of menarche (years)	14.5 ± 1.4	14.1 ± 1.4	0.14
Age at first surgery (years)	31.7 ± 5.6	30.7 ± 5.7	0.32
BMI (kg/m2)	20.8 ± 2.3	21.1 ± 2.8	0.51
Gravida			0.64
0	30.4 (14)	34.3 (36)	
≥1	69.6 (32)	65.7 (69)	
Parity			0.81
0	63.0 (29)	61.0 (64)	
≥1	37.0 (17)	39.0 (41)	
Dysmenorrhea			0.15
No	47.8 (22)	35.2 (37)	
Mild or moderate	52.2 (24)	64.8 (68)	
Uterine retroversion			<0.01
No	54.3 (25)	80.0 (84)	
Yes	45.7 (21)	20.0 (21)	
CA125	67.9 (20–1009)	63.0 (8.4–337.2)	0.85
Stage			0.76
I, II, III	91.3 (42)	92.4 (97)	
IV	8.7 (4)	7.6 (8)	
Laterality			0.78
Bilateral	34.8 (16)	37.1 (39)	
Unilateral	65.2 (30)	62.9 (66)	
Cyst diameter (cm)	5.5 ± 2	5.8 ± 2.2	0.43
Follow-up time (months)	82.7 ± 36.6	51.0 ± 33.7	<0.01
Postoperative medical treatment duration			0.40
< 3 month	19.6 (9)	17.1 (18)	
3–6 month	34.8 (16)	25.7 (27)	
≥ 6 month	45.6 (21)	57.2 (60)	
Postoperative medical therapies			0.47
GnRHa	56.5 (26)	66.7 (70)	
LNG-IUS/ (LNG-IUS + GnRHa)	8.7 (4)	2.9 (3)	
Progestin	15.2 (7)	12.4 (13)	
COC	8.7 (4)	10.5 (11)	
None	10.9 (5)	7.6 (8)	
Symptoms before the second procedure			0.01
D + G + I	23.9 (11)	5.7 (6)	
Dyspareunia	6.5 (3)	1.0 (1)	
GI symptoms	6.5 (3)	1.0 (1)	
Infertility	10.9 (5)	3.8 (4)	
Mass effect	23.9 (11)	35.2 (37)	
D + P	43.5 (20)	47.6 (50)	
Dysmenorrhea	41.3 (19)	45.7 (48)	
Pelvic pain	2.2 (1)	1.9 (2)	
Others	8.7 (4)	11.4 (12)	

Values are presented as mean ± standard deviation or percentage (number) or median (range).

DE, deep endometriosis; BMI, body mass index; GnRHa, gonadotropin-releasing hormone agonist; LNG-IUS, levonorgestrel-releasing intrauterine system; COC, combined oral contraceptives; D + G + I, dyspareunia + gastrointestinal symptoms + infertility; GI, gastrointestinal; D + P, dysmenorrhea + pelvic pain.

**Table 2:** Multivariate regression analysis of the risk factors for DE.

Variables	OR (95% CI)
Uterine retroversion	3.72 (1.62–8.53)
Follow-up time (≥5 years)	5.03 (2.29–11.02)

OR, odds ratio; CI, confidence interval; DE, deep endometriosis.

In the DE group, 23.9% had dyspareunia or gastrointestinal symptoms or infertility, 23.9% had mass effect, 43.5% had dysmenorrhea or pelvic pain before the second procedure, while in the non-DE group, that was 5.7%, 35.2% and 47.6%. There was a significant difference in the symptom distribution ahead of the recurrent surgery between the two groups (*p* < 0.01). Among the 46 DE patients, the description of the location of the DE lesions is shown in Table 3. The diameter of the deep lesions ranged from 0.5 cm to 4 cm. Twelve (26%) DE patients underwent hysterectomy during the second surgery, while 11 (10%) patients underwent hysterectomy in the non-DE group. During the second surgeries for DE patients, three patients were complicated with bowel rupture, one patient was complicated with bladder rupture, and another patient was complicated with cervical laceration. None of these complications occurred in the non-DE group during the second surgery.

**Table 3:** The description of the locations of the DE lesions.

Location	Number of cases involved	Percentage (%)
Uterosacral ligament	37	66
Rectosigmoid colon	10	18
Ureter	6	10
Pouch of douglas	1	2
Rectovaginal septum	1	2
Vagina	1	2

DE, deep endometriosis.

## Discussion

If PE, OE or DE lesions represent three different disease entities, it comes very naturally that they may have different pathogenesis as well as different risk factors in comparison with each other. However, whether PE, OE and DE share different risk factors remains a source of debate. Berube et al. conducted a study of 329 infertile patients with endometriosis and 262 infertile control women without endometriosis (19), it showed that a history of previous deliveries is a risk factor for the development of DE lesions but not for other types of lesions. In another study, Sangi-Haghpeykar et al. found a menstrual cycle of ≥30 days significantly increased the risk of superficial, but not deep endometriosis (20). Other studies showed that higher incidence of OC pill use for severe primary dysmenorrhea before 18 years of age, women with any siblings, gastrointestinal symptoms during menstruation, or eating a greater number of fruit/vegetables per day are risk factors for the future development of DE (21, 22). While in a study conducted by Parazzini et al., nulliparae and low body mass index are both risk factors for the development of DE as well as OE and PE, indicating that DE as well as OE and PE share similar risk factors (23). Another study conducted by Borghese et al. showed that low birth weight is independently associated with the risk of endometriosis, irrespective of phenotypes (24).

In this study, we focused on the risk factors for coexistent DE in recurrent OE patients as DE was reported more likely to occur in these patients (11). Knowing the risk factors can help us in earlier risk stratification, prevention as well as diagnosis of the DE patients. We found that uterine retroversion during the first surgery and a long follow-up time (≥5 years) were risk factors for coexistence of DE in recurrent OE patients. To the best of our knowledge, this is the first study to explore the risk factors for coexistent DE in recurrent OE patients.

Our study indicates that uterine retroversion during the first surgery of OE may facilitate DE formation. Endometriosis is considered as an inflammatory and adhesiogenic disease. Ott et al. reported that patients with a retroverted uterus as well as pelvic pain could be successfully treated by laparoscopic uterine suspension to reduce adhesion formation, suggesting the potential role of uterine retroversion on adhesion formation (25). Seracchioli et al. reported 42 patients with uterine retroversion and posterior DE localized in the rectovaginal septum in 15 patients and the rectum in 27 patients who underwent a hysteropexy procedure after complete laparoscopic excision of endometriosis. After 12 months of follow-up, the correction of the uterine position persisted in most of the cases, their pain symptoms were significantly improved, and no case of recurrence was observed (26). It seems that postoperative OE patients with uterine retroversion, owing to their anatomical features, are more likely to develop posterior pelvic adhesions in the colon and rectum, which may facilitate the dissemination of retrograde menstruation and the subsequent development of DE lesions. And hysteropexy with plication of round ligaments and tilting of the uterine fundus might be a reasonable option to prevent the DE formation in some selected patients.

We also found that a long follow-up time (≥5 years) is another risk factor for coexistent DE in recurrent OE patients. In our study, for recurrent patients with a long follow-up time, the probability of developing DE was almost three times that of the other patients, and approximately 30% (46 out of 151) of our patients developed DE at their recurrent surgery, which is similar to a recently published article by Nirgianakis et al. (27). In their study, one hundred and twenty-four patients were diagnosed with OE during the primary surgery, among which 39.5% of these patients subsequently presented with DE lesions in their recurrent surgery, and the median time to first recurrence surgery with OE was 27 months, while for that of DE it was 51 months. However, they did not draw a conclusion regarding whether there was a significant difference in the follow-up time between the two groups. Our study suggested that for recurrent OE patients with a follow-up time of at least 5 years, coexisting DE lesions should be considered, and a careful examination should be performed before conducting a second surgery.

Postoperative medical treatment, especially long-term hormonal treatment plays an important role in the prevention of recurrent endometriosis (28, 29). However, in our study, there was no significant difference in terms of postoperative treatment duration or postoperative medical treatment options between the DE and non-DE groups, indicating that these postoperative treatment regimens have similar effects on prevention of both OE and DE lesions, and long-term hormonal treatment should be encouraged.

Several studies have probed the mechanism of recurrence in endometriosis (30–32). Recurrent lesions could originate from either incompletely removed or inadequately treated lesions. Vignali et al. reported that DE lesions reappeared at recurrent surgery in the same area of the pelvis involved in the first operation (33). In another study reported by Exacoustos et al*.* including 62 patients with recurrent OE, 80.6% of patients had recurrence on the treated ovary (34). In addition, *de novo* lesions derived from dissemination by retrograde menstruation, lymphovascular invasion by endometriotic foci as well as immunological factors could also be involved in recurrence (30). As unrecognized deep lesions may be neglected during the primary surgery and previous studies have already reported that several factors are associated with coexisting DE lesions in OE patients (11–13), we set strict criteria when selecting patients. Patients with deep dyspareunia, severe gastrointestinal symptoms, severe dysmenorrhea, infertility, cul-de-sac obliteration and adenomyosis during the first surgery were all excluded from this study. If there were no unrecognized deep lesions during the first surgery, then the development of DE lesions in recurrent OE surgery should more reasonably be considered as *de novo* lesions.

Another controversial issue is whether endometriosis should be considered a progressive disease. Koninckx et al. reported a 3-year prospective study of 643 consecutive laparoscopies for endometriosis patients and suggested that endometriosis is a progressive disease (35). Unger et al. reported a case series of adolescent patients with complaints of severe pelvic pain who were diagnosed with stage 1 endometriosis at the time of first laparoscopy, with a two- to five-year follow-up, a second laparoscopy revealed that each patient's disease had progressed to a higher stage (36). In contrast, Fedele et al. reported that in some untreated patients, second-look laparoscopy showed approximately 40% of cases with no variation in the lesions and even regression of some of the peritoneal implants (37). While some DE lesions were not found to be progressive, even with a follow-up of 10 years (38). Still, it is uncertain that whether there is an evolution among lesion subtypes, some authors argued that progression from PE to OE or DE or from OE to DE lesions had never been observed (39). Our study advocates that endometriosis is a progressive disease and quite a few OE patients can recur and progress toward a more severe subtype.

Our study may have some limitations. For a precise definition of endometriosis subtypes, we made a diagnosis based on the patients' history, physical examination, laboratory tests, radiological imaging, and intraoperative and histological findings, and in particular, we set strict criteria to exclude DE patients during the first surgery. However, recurrent patients without clinical symptoms or under effective medical treatments who do not request a second surgery were not included in this study. Therefore, some selection bias may exist in our study. Further studies to include all recurrent patients would be interesting, but diagnosing DE without surgical intervention would introduce more challenges. In addition, there might be other risk factors that we did not consider. For example, according to a recent study by Dai et al. (22), other factors, such as dietary habits, may have an impact on the development of endometriosis subtypes, we lack these factors in our study. Finally, this is a single-center study, further multicenter research is required to consolidate the findings in our study.

## Conclusion

Our study suggests that for recurrent OE patients, uterine retroversion during the first surgery and a long follow-up time are risk factors for the development of coexisting DE, early prevention and full preparation before the second surgery should be emphasized in these conditions.

## Data Availability

The original contributions presented in the study are included in the article/Supplementary Material, further inquiries can be directed to the corresponding author/s.
